# Notes and News

**Published:** 1893-08-05

**Authors:** 


### Notes and News.

Collected and Communicated.

The St. John Ambulance Brigade have received special thanks from the Home Secretary for their good services on the occasion of the Royal wedding. No less than 1,544 cases were relieved.

In spite of the apprehension felt that the Birmingham Hospital Saturday collection would not be as large this year as last, an increase of £8 is recorded. The Fund, besides distributing large sums to each medical charity, has been able to transfer £1,000 to the Moseley Convalescent Home and £250 for the District Nursing Society. The Queen's Hospital received £1,668 and the General Hospital £2,902.

The welfare of female medical students at Edinburgh is again in danger. After October 1 next the management announce that the doors of the Royal Infirmary will be closed to female clinical students. The argument is that the Infirmary cannot afford to maintain the number of beds required for this teaching. Meanwhile, Leith Hospital, the alternative place of instruction suggested, has been rejected as a qualifying institution by the triple board. It appears that the Infirmary has a building available for the accommodation of further beds, and the Scottish Association for the Medical Education of Women are, it appears, willing to equip this. The management, however, do not entertain the idea of maintaining these extra beds, and the progress of female medical education in Edinburgh is, therefore, seriously threatened. Although Edinburgh has harboured some of the most successful female students of medicine, it has a never to be forgotten history of repression towards the women pioneers who sought its teaching. It is to be hoped that the spirit of progress in civilisation is not on the decline in Edinburgh, and that we shall soon learn that Edinburgh is not behindhand in furthering the progress of female medical students, and that the half promise that the management will reconsider the matter may be carried out after all.

The ninth annual meeting of The Hospitals Association is to be held at St. Thomas's Hospital, on Friday, 4th inst., at five p.m.

Lady Cobham opened the Corbett Hospital at Stourbridge, the gift of Mr. John Corbett, M.P., to his native town. The gift includes the mansion standing in an estate of 30 acres, and a sum of £5,000 as endowment.

The annual report of the British Medical Association shows that the inquiry into the complaint that Irish and Scotch diplomates were debarred from election to appointments at medical charities has resulted in the following facts being elicited—that out of 270 hospitals 17 only have definite rules excluding such canditates, and 187 have no restriction at all.

The Camberwell parish authorities do not apparently bear a very good character for management. At a recent coroner's inquest on the body of a little boy of that parish found drowned, the Coroner severely reprimanded those responsible for the facts elicited. The child was placed in the mortuary without covering, and no disinfectants were used. The local authorities repudiate the responsibility, and, as is not infrequently the case in these days of multiple governments, the public must suffer considerably before a remedy is found.

The proposal of the Metropolitan Asylums Board to erect an infectious hospital at Norwood has met with the usual suburban opposition A correspondent to the *Globe* suggests that such hospitals for the metropolis should be erected some 30 or 40 miles from London, in some sparsely inhabited locality, and that the Board should have its own branch line if necessary and own train service. He suggests that the cost of such a proceeding would be no greater than the purchase of the proposed costly sites. If anything could prove an effective preventive we should imagine this prospect of travelling in sickness would certainly be it.

Mr. Howgrave Graham, well-known as the energetic secretary of the Hospital for Epilepsy and Paralysis, Regent's Park, is bringing out a new setting of Tennyson's "Break, Break, Break," composed by himself, the proceeds of the sale of which are to benefit the hospital. The beautiful poem has attracted many composers, who have mostly treated it in a more or less descriptive and florid style. Mr. Howgrave Graham seems to have grasped this fact, and the rich harmonies of his composition in the minor, accord with the pathetic words of the song, and will appeal to many who have as yet been dissatisfied with the musical setting of these favourite words. We hope Mr. Howgrave Graham's composition will have a large sale, as the modest expenditure of two shillings will bring gain both to the hospital and the purchaser. The composer has every claim to a wider reputation, as his previous efforts also prove that he has a just title to be regarded as a musician. Orders for copies should be sent to the Secretary, at the hospital.

